# Why Counseling Intervention Fails to Improve Compliance towards Antiretroviral Therapy: Findings from a Mixed-Methods Study among People Living with HIV in Bali Province, Indonesia

**DOI:** 10.3390/idr13010015

**Published:** 2021-02-05

**Authors:** Anak Agung Sagung Sawitri, I Nyoman Sutarsa, Ketut Tuti Parwati Merati, I Made Bakta, Dewa Nyoman Wirawan

**Affiliations:** 1 Department of Public Health and Preventive Medicine, Faculty of Medicine, Udayana University, Jalan PB Sudirman, Denpasar, Bali 80223, Indonesia; sutarsa.nyoman@anu.edu.au (IN.S.); nym_wirawan@unud.ac.id (D.N.W.); 2 Rural Clinical School, ANU Medical School, ANU College of Health & Medicine, The Australian National University, Florey Building #54, Mills Road, Canberra, ACT 0200, Australia; 3 Department of Internal Medicine, Faculty of Medicine, Udayana University/Sanglah General Hospital, Jalan Pulau Nias, Denpasar, Bali 80232, Indonesia; parwati_merati@unud.ac.id (K.T.P.M.); bakta@unud.ac.id (IM.B.)

**Keywords:** mixed-methods, counseling, HIV/AIDS, acceptance, adherence

## Abstract

This study explores the contribution of counseling to improving acceptance of and adherence to anti-retroviral therapy (ART) among people living with HIV (PLHIV) and identifies key issues associated with its implementation. We conducted a longitudinal mixed-methods study in Bali Province between 2015 and 2017. The study participants were 170 newly diagnosed PLHIV and 17 outreach-counselor workers (OWs). We interviewed PLHIV for their experiences in receiving counseling, and acceptance of and adherence to ART. We surveyed four counseling domains (privacy, contents, frequency, and duration) and explored the key findings through in-depth interviews. In addition, 24 exit interviews and record reviews were performed. Quantitative data were analyzed descriptively, and qualitative data were analyzed thematically. Out of 170 PLHIV who received the counseling program, 139 (81.76%) accepted ART, and 52 (37.4%) discontinued ART in six months of follow up. Although counselors covered most of the content (16/17 components), the median time and frequency of counseling were insufficient. Despite a high score of HIV counseling provided to PLHIV in our study location, the overall acceptance of and adherence to ART remains fair or moderate. Our study suggests that counseling before ART initiation is inadequate for improving acceptance and adherence to ART in Bali Province. This reduced effectiveness is influenced by internal issues (interpersonal skills, limited technical capacity) and external factors both from PLHIV and society (stigma, disclosure, discrimination).

## 1. Introduction

HIV/AIDS remains a public health challenge in Indonesia with 524,371 cases cumulatively in June 2020 [1]. The HIV epidemic in Indonesia (other than Papua) is concentrated in key populations, particularly among female sex workers (FSWs) and men who have sex with men (MSM) [1,2]. The national prevalence among adult populations is 0.27%, with newly infected cases of 28,842 in 2019 [3]. In 2014, UNAIDS announced the 90–90–90 global target to end the HIV epidemic by 2030: 90% of all PLHIV should know their status; 90% of PLHIV should have access to ART; and 90% of those on ART should have an undetectable viral load. Despite various intervention strategies, Indonesia reported the fast-track global target of only 35–36–NA in 2017 [4], which is lower than Thailand and Cambodia [4]. To end the HIV epidemic by 2030, Indonesia needs to provide equitable access to HIV prevention, care, and treatment to the most-at-risk populations regardless of geographical and socio-economic locations.

AIDS mortality has been decreased since 2000 due to improved access to ART [1,5]. ART suppresses HIV viral load [6] and can prolonged life, improve health and quality of life, and reduce risks of HIV transmission [6]. This fact signals the needs for comprehensive and sustainable services for HIV prevention, care, and treatment [6,7]. 

Indonesia has implemented the continuum and comprehensive health services for HIV/AIDS since 2013, which consists of five key strategies, including a counseling program, to improve acceptance of and adherence to ART [8]. In addition, the Ministry of Health (MOH) of Indonesia has promoted early ART since September 2013 [9]. The roles of HIV counsellors are central for promoting early ART initiation and treatment adherence. The national HIV counseling and testing guideline [10] recommends pre- and post-test counseling for a client. The pre-test counseling aims at preparing clients for HIV testing, while post-test counseling aims at adapting clients for the test result. Counseling must be provided by trained/certified healthcare providers. Counseling training for healthcare staff is administered by the MOH using the national module (called *Modul Pelatihan Konseling dan Tes*) and delivered by accredited counseling trainers [10].

There are five key elements of HIV counseling: informed consent, confidentiality, counseling, accurate testing and linkage to prevention, care, and treatment services [10]. More specifically, in post-test counseling, HIV status must be revealed in person, as early as possible, through a brief and clear message; and the management plan must be discussed, which includes prevention strategies from HIV and other sexually transmitted infections (STIs) and risks of HIV re-infection, further treatment, and care to improve quality of life and general health status. It has to be done by the same person who provides the pre-test counseling. Furthermore, adherence counseling is provided to clients who decided to start ART. It includes information related to the drugs, treatment failure, the importance of adherence, access to ART, and monitoring of side effects.

However, previous surveys among key population groups in Indonesia found that the coverage of early ART and adherence rates remain limited [11,12]. Until recently, there have been very few studies exploring the roles of the counselling program in improving acceptance of and adherence to ART [13,14]. Some studies identified that the inadequacy of a counseling program is contributed by burn-out and impractical protocol [15], short duration and lack of frequency [16], and no systematic screening to evaluate clients’ motivations [16,17,18,19]. Most of the previous studies are not specifically targeting key population groups, are cross-sectional in nature, and are inconclusive. Our current study is the first longitudinal study exploring the contribution of counseling intervention in improving acceptance of and adherence to ART. In this study, we reported how counseling after HIV diagnosis and the first three months of ART initiation contributes to the acceptance of and adherence to ART among newly diagnosed HIV patients in a private non-government organization (NGO) clinic in Bali Province, Indonesia. 

## 2. Materials and Methods

The study was conducted at the WM Medika Clinic of the Kerti Praja Foundation (KPF). KPF provides HIV/AIDS and other STI prevention, care, and treatment programs, particularly to marginalized groups such as FSW, MSM, people who inject drugs (PWIDs), and transgender. The outreach workers (OWs) of KPF perform outreach activities, provide information about HIV testing, and offer HIV counseling and testing. They also provide pre and post-test counseling for clients. HIV-positive clients who wish to initiate ART will be referred to the doctor for comprehensive HIV and ART services. 

A concurrent mixed-methods study by combining quantitative and qualitative approaches was conducted. The quantitative element involved a cohort of newly diagnosed HIV patients from 15 October 2015 to 31 January 2017. As part of their usual roles, OWs administered pre- and post-test counseling to PLHIV and offered early ART initiation. OWs advertised the study and recruited potential study participants. Patients were given a maximum of a three month period to decide whether to accept or decline the treatment [19]. The last date of observation for ART acceptance was on 24 September 2016. Subsequent observations were made only to those who accepted ART, and they were followed for about six months to measure their adherence towards ART. Interviews with PLHIV to gather information regarding their experiences with counseling services were carried out in two occasions: immediately after the establishment of HIV diagnosis/status and three months after the ART initiation date. 

Study participants were newly diagnosed HIV patients at the WM Medika Clinic. We calculated the sample size to compare acceptance of and adherence to ART based on counseling scores using the 95% confidence level, power of 80%, proportion of PLHIV on ART and poorly treated by doctors (38%) [20], drop out estimation of 5%, and a relative risk of 2. PLHIV aged > 17 years-old and agreement to participate in the study were consecutively selected from the clinic. Key informants for in-depth interviews included 5 PLHIV (2 FSW, 2 MSM, and 1 non-key population) who discontinued ART, 3 PLHIV who maintained ART for at least a year (2 MSM and 1 FSW), and 2 OWs (1 counselor for FSW and 1 for MSM). A total of 10 informants were determined as a base-line. However, given the diverse backgrounds of our key informants, it allowed rich data to be collected; and data saturation was reached from these 10 key informants. 

Data were collected using a pre-tested structured questionnaire and an interview guide. The survey was administered by trained enumerators consisted of 17 OWs and three research assistants. OWs who provided counseling for PLHIV were appointed as interviewers for the convenience of PLHIV (T-1). Trained and independent research assistants were responsible for the second interview (T-2). Information regarding patient experiences during pre- and post-test counseling was explored in the second interview. These patients were on ART between 46 and 168 days after the initiation (median 89, IQR 26). PLHIV were followed for six months, and their adherence to ART was recorded. All in-depth interviews were conducted directly by the researcher using the local language (Indonesia and/or Javanese/Balinese). Only selected quotes were translated into English. 

The quantitative variables for this study included counseling experience, ART acceptance, and ART adherence among PLHIV. The National HIV Counseling Guidebook outlines key components of the HIV counseling and testing program [10]. We categorized acceptance of ART into “accept” when PLHIV started ART after the counseling program, and “not accept” when PLHIV refused ART or if PLHIV did not responding to the ART offer within three months after their HIV diagnosis. Similarly, we classified adherence to ART into “adhere” and “not adhere”; where “not adhere” was defined when PLHIV did not access the treatment as prescribed and/or were declared lost to follow-up by the field staff. Additionally, we explored socio-demographic characteristics and the history of counseling training. For the qualitative component, we explored knowledge and perception of OWs about early ART initiation, their perceived roles and experiences if patients presented with side effects from the treatment, and key issues in providing quality counseling including personal capacity and skills, interpersonal relationships, and associated psychological burden.

Quantitative variables were analyzed descriptively using computer software (STATA 12, StataCorp LLC, College Station, TX, USA). We compared socio-demographic characteristics of PLHIV at the study initiation (T1) and at three months on ART (T2) using chi-square for categorical variable and median test for interval variable. OW characteristics were presented descriptively. Counseling activities were calculated as percentage and compared using two proportion tests at the T1 and T2. Counseling substance’s score, length, and frequency of counseling were compared in terms of minimum, maximum, and median score, and IQR using median tests. We categorized counseling score (substance, time, frequency) into upper and same/lower compared to the median. We measured contribution of counseling program towards acceptance of and adherence to ART using the Fischer exact test for cohorts. Qualitative data were analyzed using thematic analysis and presented according to key themes identified from the data. Both qualitative and quantitative data were carefully examined to develop explanations of the observed findings.

## 3. Results

**Figure 1:**
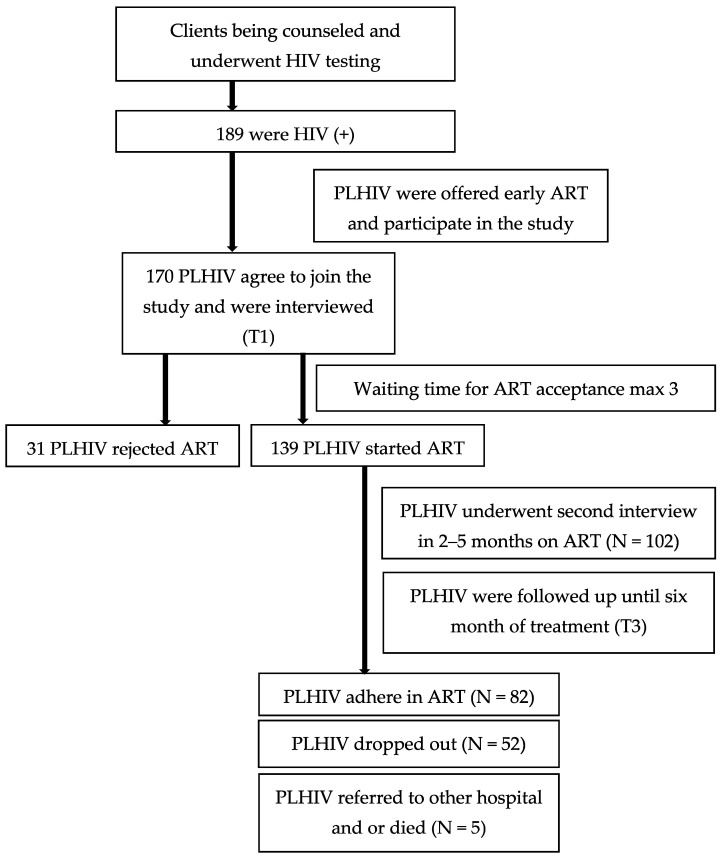
Study flowchart.

A total of 189 newly diagnosed PLHIV were offered an early ART initiation and were invited to participate in the study. A total of 170 (89.9%) agreed to participate in the study, and 139 PLHIV (81%) PLHIV accepted the treatment within 0–97 days (median = 7, IQR = 3). After six months on ART, 37.4% of PLHIV discontinued their ART at the first month (12 patients), second month (9 patients), third month (8 patients), fourth month (12 patients), fifth month (7 patients), and sixth month (4 patients). Additionally, five patients were referred to hospitals and/or died (Figure 1).

### 3.1. Socio-Demographics Characteristic of Respondent

Table 1 shows socio-demographic characteristics of PLHIV who agreed to participate in the study and those who accepted ART three months after the counseling. Table 1 also depicts socio-demographics of 17 OWs who participated in the study. Socio-demographic characteristics between PLHIV who agreed to participate in the study and those who finally accepted the treatment were similar. OWs were predominantly male, mean age 31 years old, high school or tertiary education graduates, and with working period of 3 years.

### 3.2. Counseling Activities Reported by People Living with HIV (PLHIV)

Table 2 depicts an evaluation of counseling activities received by PLHIV. In the first interview, the coverage for each counseling item was very high (above 90%), except for the privacy component, where 11% of PLHIV reported that someone else was present in the room during the session. About half of PLHIV reported the duration was less than 30 min prior to initiating ART. In the second interview, we found that the overall counseling score was very high; however, some counseling items were decreased in quality (e.g., understanding of HIV testing result and explanation about HIV transmission, prevention, and ART). PLHIV in the second interview reported less privacy breach in comparison to the first interview. Almost half of PLHIV reported that they received more frequent counseling but shorter in duration (about 10 min). In the first interview (T1), 170 PLHIV were interviewed; however, for the second interview (T2), only 102 PLHIV were interviewed due to various reasons: dropped-out from the study, non-compliance, ARV drugs were supplied for 2–3 months for those who live far away from the clinic, and migrated to other areas and family members collected the ARV on behalf of the patients.

### 3.3. Correlation between Counseling and Acceptance and Adherence to Antiretroviral

Table 3 shows that there was no significant association between total score, time, and frequency of counseling to the acceptance of and adherence to ART at six months follow up.

### 3.4. Findings from Qualitative Result

#### 3.4.1. Summary of In-Depth Interviews with Counselors and PLHIV

A total of 10 in-depth interviews were conducted, which included eight PLHIV and two OWs. We identified positive attributes of the counseling program: ongoing support from OWs to transfer knowledge about HIV/AIDS and ART, reaching out to maintain contact and communication with PLHIV, reminding PLHIV to take ART regularly and come to the clinic for routine medical monitoring, and providing social support and care when PLHIV encountered problems related to their treatment. These can be seen in the following quotes:

“All have been explained… feel gap… not like being with your friends, you can tell more… feeling comfortable… I received both information and support from here… ”

(Subject-1, MSM)

“I remember to consume the drug…… [OWs] remind me to take my medication regularly through text message…… ”

(Subject 2, FSW)

“… They [OWs] advised me to return to the clinic but I cannot go back, … I told [them/OWs] that I suffered from side effects … looks tiny and sick.”

(Subject-3, MSM)

Despite these positive attributes, we found many challenges leading to reduced efficacy or effectiveness of counseling in improving acceptance of and adherence to ART. Firstly, many PLHIV reported that the quality of counseling does not always meet their expectations, mainly due to inadequate capacity or skills of OWs. These can be seen in the following quotes:

“Yes, it contains lots of information, but I could not really get the idea… The counseling was very short… about ten minutes…”

(Subject-3, MSM)

“… [OW] told me not to open my status to every one…”

(Subject-4, FSW)

Secondly, limited skills and capacity of OW have led to reduced clarity, lack of understanding, and misperception among PLHIV regarding their HIV status, processes of initiating ART, and functions of ART as treatment and prevention, as shown in the following quotes:

“… [OW] not talked about ART processes or its functions as both prevention and treatment… he told me to take my medication for my own health. I did because I want to be healthy…”

(Subject-3, MSM)

“… I thought if I am not working in sex work, I could not ask for ART from the clinic”

(Subject-4, FSW)

“… I don’t remember anything from the counseling… ”

(Subject-2, FSW)

Thirdly, skills and approaches of OWs are varied and highly influenced by their intra and interpersonal skills. This leads to variations of counseling techniques and the overall performance or effectiveness of the counseling. Specifically, PLHIV reported that information about ART and its side effects, disclosure, and stigma are rarely discussed during the counseling. OWs expressed that a high workload and external factors such as stigma and discrimination from society have influenced the overall efficacy of the counseling program. These can be seen in the following quotes:

“I never been told about that [stigma and disclosure] … No, the doctor also never discussed that [stigma and disclosure] with me”

(Subject-1, MSM)

“No, I cannot tell him…, I looked for more information out there. I have been on herbal medication for one month, while he [OWs counselor] didn’t know… ”

(Subject-1, MSM)

“Previously I was sure [to determine whether PLHIV will adhere to ART or not before initiate treatment])… finally, I realized that I commonly make mistake… some people who have long speech and looks smart but finally not adhere to treatment… some people look stupid and accept, but they have strong motivation and adhere to the treatment… ”

(Subject-10, OW)

#### 3.4.2. Summary of Exit Interviews to PLHIV and Outreach Counselor Workers

We also conducted 24 exit interviews with PLHIV at three months of ART. We found that OWs provided less (30–45%) general information about HIV transmission or prevention, including condom use, in comparison to information about ART (side effects, managing side effects, adherence, and reminder for control) (55–100%). Only one (4.5%) PLHIV discussed disclosure issues with OWs. Moreover, our exit interviews revealed that the frequency of communication by text messages between OWs and PLHIV during the first three months of treatment were varied from none to one time (33.33%), and by phone calls ranging from none to one time only (79.17%). Through further interviews with 17 OWs, we found about half (8 OWs) were able to reach around 20 PLHIV, had to counsel one potential client per day, and had to maintain communication (face-to-face or text message or phone call) with three PLHIV on ART per month. In terms of training activity, half of OWs (8) had received an average of 4.5 days of national counseling training; however, some of this training occurred more than five years ago. Few OWs had also received refresher training (2), couples counseling training (2), positive prevention training (2), and overcome burn out training (1).

## 4. Discussion

This study found that HIV counseling does not improve acceptance of and adherence to ART among PLHIV in our study location. We argue that this reduced effectiveness is influenced by internal factors of OWs (e.g., intra and interpersonal skills) and external factors both from PLHIV and societies (e.g., stigma, disclosure, and discrimination). 

We identified that OWs have provided key information related to HIV and ART to PLHIV; however, our qualitative study revealed a huge gap between information provided by OWs and the amount of information being captured and retained by PLHIV three months after ART initiation. We observed lack of understanding and misperceptions among PLHIV about HIV and ART. When PLHIV decide to commence treatment, they are at different stages of readiness [21,22,23] for which they might not be able to absorb/retain more new information, especially when it is provided in a relatively short duration with infrequent enhancements. It is unfortunately beyond our study to assess the true readiness of PLHIV for receiving ART. 

Implementation of an early ART initiation strategy requires counsellors to be able to motivate clients for positive behavior changes within a short-period of time [23,24], which consequently will improve acceptance, willingness, and readiness to initiate early ART [22,25]. After PLHIV started ART, the main focus of counseling was shifted toward improving compliance to treatment. Our study found that the counseling was lacking in both frequency and duration. Similarly, the depth of information provided to PLHIV about ART and overall counseling skills were lacking. These limitations have been identified by many scholars as key barriers to successful implementation of counseling programs [16,18,19]. Another study suggests the importance of a pre-screening interview to assess PLHIV who are better prepared to initiate early ART [25]. This constitutes a gap in the current ART counseling practices within Indonesian contexts because positive perceptions need to be maintained, increased, and continued to promote more stable health behaviors [23]. HIV counseling and testing is expected to contribute to improving the level of knowledge and positive perceptions about ART, leading to positive attitudes towards ART, increased willingness to take medication, and improved compliance towards treatment [26].

Although most OWs in our study had received five days of the HIV voluntary counseling and testing (VCT) training program following the designated module of MOH of Indonesia [10], only a few of them had participated in refresher training. Lack of refresher training opportunities has been identified in the literature as a key contributing factor to lower quality counseling service [27,28,29]. In addition, lack of intensity and frequency of trainings [28,29] and inadequate training materials [28,30,31] contribute to the overall quality of counseling provided by OWs. Ineffective ART counseling is contributed by limited training opportunities for specific skills: ART [31], handling acute stress at HIV diagnosis [31,32] and screening of self-efficacy of PLHIV [17]. Several external factors such as increased demands from funding agencies for meeting targets [15,33] and stigma/discrimination from the society [34,35,36,37] can also lead to ineffective counseling. With limited opportunities for refresher training, as well as combinations of various external barriers, OWs face ongoing challenges in providing continuous support to PLHIV on ART for at least six months [38]. Beyond counselor capacity, the availability of long-term ARV injection [39,40] might improve the acceptance of and adherence to ART. A recent study has shown high acceptability among PLHIV and those who are at risk of HIV [41]; however, this method is not yet available in Indonesia or Bali Province. 

Creating support systems for OWs, including opportunities to refresh training and to create specific training programs, is vital in order to improve competence and self-efficacy when providing counseling to PLHIV [32]. In addition, OWs need to be assisted in determining PLHIV at risk for ART drop-out, for example through PLHIV self-efficacy assessments, so that referrals to appropriate health providers can be made, or tailored programs using a multidisciplinary model can be created to assist PLHIV in maintaining positive attitudes towards ART [7,42,43,44,45].

Previous studies have identified socio-demographic characteristics that contribute to ART drop out, namely younger age [46,47,48], more clinical symptoms [17,49,50], FSW status [34,51,52], and MSM [36]—although their association is not always consistent [48,50]. These findings are essential to facilitate OWs in developing appropriate counseling strategies tailored to specific needs or characteristics of PLHIV. These conditions are beyond the scope of our study, and further study is warranted, especially from an Indonesian context. 

Using a combination of quantitative and qualitative methods, our study allows for comprehensive observation and an in-depth picture about counseling practices and experiences from OW and PLHIV perspectives, and their contributions towards acceptance of and adherence to treatment. The fact that interviews were performed by OWs at the beginning of the observation could not exclude the possibility of social desirability responses from PLHIV, for which they provide answers that are expected by the interviewer. In addition, participants for our study were selected using consecutive sampling; as such, our study might suffer from potential selection bias and weaknesses in generalization. 

Despite a high score of HIV counseling provided to PLHIV, the overall acceptance of ART remains fair or moderate. This study confirmed that HIV counseling prior to testing and during treatment does not improve the overall adherence towards ART. 

## Figures and Tables

**Table 1:** Characteristics of PLHIV and OWs.

Characteristics		PLHIV Agreed to Participate in the Study (*n* = 170)		PLHIV Accepted ART Three Months after the Counseling (*n* = 139)		*p* *
		*n*	%	*n*	%	
**PLHIV**						
**Sex**	Female	55	(32.4)	43	(30.9)	0.78
	Male	115	(67.7)	96	(69.1)	
**Age (year)**	Median (IQR)	30.1	(25.2–36.1)	29.7	(25.0–39.9)	0.93
**Education**	Illiterate–elementary school	35	(20.6)	25	(18.0)	0.56
	Junior high school	25	(14.7)	22	(15.8)	0.79
	High school and University	110	(64.7)	92	(66.2)	
**Occupation**	Unemployed	23	(13.5)	19	(14.0)	0.73
	Employed	147	(86.7)	120	(86.0)	
**Salary (million, IDR** = Indonesia Rupiah)	Median (IQR)	2.0	(1.5–3.0)	2.0	(1.6–3.1)	
**Work length (year)**	Median (IQR)	2.0	(1.0–4.0)	2.0	(1.0–4.0)	
**Marital status**	Not married	97	(57.1)	83	(59.7)	0.64
	Married	28	(16.5)	23	(16.6)	0.96
	Widow/divorce	45	(26.5)	33	(23.8)	
**Risk group**	Heterosexual (include housewives)	27	(15.9)	25	(18.0)	0.63
	FSW	45	(26.5)	33	(23.7)	0.58
	MSM, bi-sex, transgender	98	(57.7)	81	(58.3)	
**Outreach workers (*n* = 17)**						
**Sex**	Female	5	(29.0)			
	Male	12	(71.0)			
**Education**	High School	10	(58.9)			
	University	7	(41.1)			
**Age (year)**	Median (IQR)	36.0	(33.8–43.0)			
**Work length (year)**	Median (IQR)	3	(2–5.3)			

Note: * tested by chi square and median test.

**Table 2:** Percentage of activities of counseling based on PLHIV evaluation after HIV diagnosis and two to five months after ART initiation.

Counseling Item	T1 (*n* = 170)		T2 (*n* = 102)		*p* *
	Yes		Yes		
	*n*	%	*n*	%	
Counselor is the OW	156	(91.8)	92	(90.2)	0.63
There is someone else during counseling	19	(11.2)	20	(19.6)	0.06
Counseling is in private room	168	(98.8)	101	(99.6)	0.49
Explained for test result	169	(99.4)	101	(99.6)	0.83
Understand the explanation of test result	167	(98.2)	95	(93.1)	0.03
Explained risk of HIV transmission	170	(100)	95	(93.1)	0.00
Explained on preventing transmission	170	(100)	97	(95.1)	0.05
Explained further laboratory examination	168	(98.8)	92	(90.2)	0.00
Offered ART	170	(100)	96	(94.1)	0.00
Explained benefit of ART	170	(100)	100	(98.0)	0.09
Explained of ways to consume ART	168	(98.8)	102	(100)	0.22
Explained about overcoming side effect	165	(97.1)	96	(94.1)	0.22
Given mobile number of OW	166	(97.6)	100	(98.0)	0.83
Given opportunity to ask and express feeling	169	(99.4)	97	(95.1)	0.02
Given opportunity to discuss	167	(98.2)	97	(95.1)	0.15
Given opportunity to think	169	(99.4)	101	(99.0)	0.72
Feel to be supported	170	(100)	102	(100)	1.00
Score counseling (1–17)					
▪ min–max score	12–17		11–17		
▪ Median score and IQR	16 (0)		16 (1)		1.00
Length of counseling (minute)					
▪ min–max minute	10–80		0–60		
▪ Median time and IQR	30 (0)		10 (15)		0.00
Frequency of counseling					
▪ min–max frequency	1–4		0–15		
▪ Median frequency and IQR	1 (1)		3 (2)		0.00

* Tested by two proportion tests.

**Table 3:** Analysis of counseling to ART acceptance and ART dropout at 6 months follow up.

**Characteristics of Counseling (T1)**	**Total (170)**		**Accept (139)**		**Reject (31)**		p **
	***n***	**%**	***n***	**%**	***n***	**%**	
Median (IQR) total items	16 (0)		16 (0)		16 (0)		
>median	149	(87.6)	121	(81.2)	28	(18.9)	0.77
≤median	21	(12.4)	18	(85.7)	3	(14.3)	
Median (IQR) time	30 (37.5)		30 (0)		30 (10)		
>median	23	(23.5)	20	(87.0)	3	(13.0)	0.77
≤median	147	(86.5)	119	(81.9)	28	(19.1)	
Median (IQR) frequency	1(1)		1 (1)		1 (1)		
>median	57	(33.2)	48	(84.2)	9	(15.8)	0.68
≤median	113	(66.8)	91	(80.5)	22	(19.5)	
**Characteristics of counseling (T1)**	**Total (134) ^a^**		**Adhere (82)**		**Dropout (52)**		***p*** **
	***n***	**%**	***n***	**%**	***n***	**%**	
Median (IQR) total items	16 (0)		16 (0)		16 (0)		
>median	116	(86.6)	71	(61.2)	45	(38.8)	1.00
≤median	18	(13.4)	11	(61.1)	7	(38.9)	
Median (IQR) time	30 (0)		30 (0)		30 (0)		
>median	20	(14.9)	15	(75.0)	5	(25.0)	0.22
≤median	114	(85.1)	67	(58.8)	47	(41.2)	
Median (IQR) frequency	1 (1)		1 (1)		1 (1)		
>median	45	(33.6)	30	(66.7)	15	(33.3)	0.45
≤median	89	(66.4)	52	(58.4)	37	(41.6)	

** Fischer-exact test; ^a^ five PLHIV have been referred to hospital and/or died.

## Data Availability

Data will be provided upon request.
